# Digital health interventions for people who use methamphetamine: a scoping review

**DOI:** 10.3389/fpsyt.2025.1658021

**Published:** 2026-01-20

**Authors:** Kathryn Fletcher, Jack Freestone, Brendan Clifford, Nadine Ezard, Stephanie Kershaw, Liam S. Acheson, Theodora Karavasilis, Krista J. Siefried

**Affiliations:** 1 The National Centre for Clinical Research on Emerging Drugs, University of New South Wales, Sydney, NSW, Australia; 2 St Vincent’s Hospital Alcohol and Drug Service, Sydney, NSW, Australia; 3 The National Drug and Alcohol Research Centre, University of New South Wales, Sydney, NSW, Australia; 4 Drug and Alcohol Clinical Research and Improvement Network, NSW Health, Sydney, NSW, Australia; 5 The Matilda Centre for Research in Mental Health and Substance Use, The University of Sydney, Sydney, NSW, Australia

**Keywords:** digital health, intervention, methamphetamine, scoping review, acceptability and feasibility, clinical impact

## Abstract

**Introduction:**

Methamphetamine use is increasing and is associated with substantial health, social and economic harms. Despite these harms, many individuals delay or avoid treatment due to personal and systemic barriers. Digital health interventions (DHIs) are a promising avenue for expanding treatment access, however their application for methamphetamine use remains relatively underexplored. This scoping review aimed to identify and synthesise existing evidence on the feasibility, acceptability, and potential clinical impact of DHIs for people who use methamphetamine.

**Methods:**

Four databases (Embase, PubMed, PsychINFO, and Scopus) were searched up to December 18, 2024. Eligible peer-reviewed studies examined DHIs aimed at reducing methamphetamine use and reported outcomes related to feasibility, acceptability, engagement, or clinical outcomes. Data were extracted and synthesised descriptively.

**Results:**

Seventeen records (from 15 studies) reporting on 1269 participants (aged ≥18) across six counties were included. Women were underrepresented (180/1,217; 14.8%) in studies reporting gender. Most participants (772/1269, 60.8%) met study inclusion criteria for methamphetamine use; the remainder (497/1269, 39.2%) met diagnostic criteria for methamphetamine use disorder. Thirteen unique DHIs were identified including web-based programs (n=2), text messaging (n=2), smartphone apps (n=6), chatbots or virtual agents (n=2), and virtual reality (n=1). Cognitive behavioural therapy approaches underpinned 8 of 13 (61.5%) interventions, and 8 of 15 studies (53.3%) used randomised controlled trial designs. Most DHIs were feasible and acceptable, with high satisfaction reported. Initial engagement was generally strong but declined over time; dropout rates ranged from 7.3% to 67.7%. Clinical impact was mixed. Some DHIs demonstrated reductions in methamphetamine use, craving or improvements in cognitive functioning and help-seeking, with some sub-groups appearing to benefit more than others.

**Discussion:**

DHIs show promise for people who use methamphetamine. While current evidence supports feasibility and acceptability, clinical outcomes are variable. High attrition and engagement challenges persist. Heterogeneity in study designs, measures, and reporting limits comparability. Future research should explore long-term outcomes, hybrid models, and co-designed approaches with a focus on gender and equity. With further development, DHIs may play a valuable role in the broader intervention landscape.

## 1 Introduction

Methamphetamine use is expanding globally. Worldwide, approximately 30 million people use amphetamine (including methamphetamine), with an upward ongoing trend in use (1). Methamphetamine is an amphetamine-type stimulant producing euphoric effects including a heightened sense of assertiveness and curiosity, increased energy, elevated interest in environmental stimuli, heightened sexual pleasure, and decreased anxiety (2). A range of harms are associated with frequent methamphetamine use, including physical and mental health impacts (3, 4), social and behavioural harms (5) and substantial socio-economic burden (1, 6), as well as methamphetamine use disorder which is associated with significant morbidity and mortality (7, 8).

While not all methamphetamine use leads to harm or disorder, some individuals experience negative consequences yet do not perceive a need for help (9). There is an estimated lag time of up to 10 years between first experiencing harms related to methamphetamine and subsequently seeking treatment for methamphetamine-related problems (10).Engaging people with substance use issues in treatment remains a challenge due to psychological, lifestyle, and treatment system factors. Several underlying, correlated factors characterise these barriers, including not believing there is a problem that requires treatment; poor social support for treatment; stigma and a fear of treatment; concerns about privacy; time demands; poor treatment availability; and difficulty gaining admission to treatment programs (11). Barriers are compounded by the time and cost demands of more traditional approaches of face-to-face or group therapy. In Australia, an estimated 25-54% of people with methamphetamine use disorder received treatment during 2022/2023 (12), highlighting a substantial treatment gap. Currently, there are no regulatory-approved pharmacological treatment options for methamphetamine use disorder. Psychosocial treatments including cognitive behavioural therapy (CBT), contingency management (CM), motivational interviewing (MI), psychodynamic therapy and mutual aid groups have been shown to reduce methamphetamine use, although these effects do not persist after the end of treatment (13).

Digital health interventions (DHIs) have the potential to transform the treatment landscape for substance use by providing accessible, personalised and effective intervention options (14). DHIs can be self-managed or clinician-assisted and offered as standalone interventions or as adjuncts (in synchronous, asynchronous, or hybrid format) to traditional care. They incorporate varying modalities including smartphone applications (mHealth), internet-based programs, text-messaging (including ecological momentary assessment), wearable sensors, chatbots and virtual reality (VR) technology. Some DHIs involve the digital adaptation of existing, evidence-based interventions (e.g. CBT, CM, MI), translating these approaches into web-based modules, apps, or automated systems. *De novo* digital interventions are purpose-built for the digital environment without direct analogues in face-to-face care, such as the S-Check app (15), that offer new modes of engagement. Other DHIs function primarily by enhancing access to traditional care, such as through telehealth platforms, appointment reminders or digital care navigation. These approaches vary both in form and in terms of their aims and outcomes, which may include encouraging help-seeking, reducing substance use, increasing engagement or retention in treatment, or addressing structural barriers to care.

DHIs targeted at reducing use and impacts of substances are still in the early stages of development, facing ongoing challenges related to device or internet accessibility, identifying suitable users, determining effective intervention strategies and delivery methods, maintaining long-term engagement, and maximizing treatment outcomes (16). Nonetheless, their potential impact is promising. As overviewed by Johannson et al. (17), meta-analyses indicate that digital interventions lead to reductions in substance use comparable to those achieved through FTF interventions. Further, those that incorporate CBT in particular are efficacious treatments for reducing alcohol and drug use overall (18). A recent scoping review identified that most DHIs for substance use to date have focused on alcohol and tobacco (17), with a paucity of interventions specifically targeting methamphetamine use. A mini-review (19) found preliminary evidence supporting technology-based interventions – both self-guided and clinician-assisted - for people who use methamphetamine. While effect sizes varied, most were small to moderate, with the strongest outcomes observed in simpler, low-intensity interventions such as text messaging, particularly among individuals not engaged in formal treatment. The review underscored the importance of tailoring these interventions to the unique needs of this population and concluded that interventions for methamphetamine are lacking. An update on this literature is now timely.

The present paper reports on a scoping review examining the acceptability, feasibility and clinical impact of DHIs for people who use methamphetamine. Two questions drive the review: (1) What DHIs are available to people who use methamphetamine? and, (2) Are DHIs for methamphetamine use feasible, acceptable, and effective?

## 2 Methods

The scoping review methodology, based on Arksey and O’Malley (20) and refined by Levac et al. (21), was deemed most suitable to map the emerging literature on diverse digital modalities, identify evidence types, highlight knowledge gaps, and inform future research directions. This approach was selected because research on DHIs for methamphetamine use is at an early stage, with diverse populations, intervention types, and outcome measures. Such heterogeneity limits the feasibility and meaningfulness of a meta-analysis, making a systematic review less appropriate at this stage. The scoping methodology allows for a comprehensive mapping of available evidence and knowledge gaps, rather than quantitative synthesis of effect sizes. The Preferred Reporting Items for Systematic Reviews and Meta-Analyses Extension for Scoping Reviews (PRISMA-ScR) guidelines (22) were followed to ensure adherence to methodological standards (see Supplementary File 1 for checklist). A protocol was not prepared or registered prior to conducting this scoping review.

The primary focus is on personal technologies (e.g., laptops, tablets, smartphones, and wearables) as they represent (1) the most effective means of expanding access to specialised information and care for individuals who are unable or unwilling to engage with traditional healthcare consistently, and (2) the most accessible and cost-efficient technology complement to conventional care (23). We included VR – even if delivered in a clinical setting – considering it as a personal technology; VR headsets are typically designed for single-user experiences, are portable, can be tailored to individual needs, are increasingly being used for personal mental health applications (24), and therefore similar to personal devices like smartphones and tablets. In this review, we adopt the definition of multi-technology interventions as using more than one technological platform which may be integrated with traditional or virtual care (23). Primary telehealth interventions were excluded as the focus here is on self-management DHIs; if the intervention had telephone calls or non-automated text messaging, a self-directed digital health component (e.g. web-based material, smartphone app) was required for inclusion in the review.

Detailed eligibility criteria are outlined in **Table 1**, broadly following the Population, Concept and Context (PCC) framework (25). Only peer-reviewed academic articles were included to strengthen the quality and validity of the results.

**Table 1:** Inclusion and exclusion criteria.

	Inclusion criteria	Exclusion criteria
Population	Individuals, groups or populations. Directly to the target group (people who use methamphetamine).	Not humans. Animal experiments, computer simulations. Indirectly via health professionals or relatives.
Concept	Digital interventions (mHealth, eHealth) delivered via Computer, Internet, Web, Telephone, Tablet, Mobile phone, Wearables, VR) that are fully or partially aimed at preventing or reducing use, risky use and addiction to methamphetamine. Multitechnology interventions (e.g. personal technologies combined with videoconferencing or phone calls). Primarily self-management interventions.	No intervention. Interventions not delivered digitally. Surveillance or monitoring of web or social media. Intervention solely involved direct contact with a health professional (whether synchronous or asynchronous) such as telemedicine/telehealth (i.e. phone, video, or other communication technology-based care). Interventions developed for conditions other than methamphetamine use/methamphetamine use disorder (e.g. depression, anxiety, other substance use disorders, polysubstance use, physical health conditions) or unrelated to methamphetamine.
Outcome	Abstinence or reduced use, risky use, addiction or harmful consequences of methamphetamine (including intentions to reduce use, cravings, improve help-seeking, improve knowledge, health, cognitive functioning, mental health and quality of life). Knowledge, attitudes or intentions in relation to methamphetamine use. Use of digital interventions (uptake, engagement, delivery and retention). Attitudes or intentions in relation to digital methamphetamine interventions. Experiences of and preferences regarding digital methamphetamine interventions (acceptability).	Not methamphetamine related. Not related to digital intervention.
Study design	Randomised controlled trials Quasi-experimental controlled trials Pilot studies Feasibility studies Non-randomised studies Qualitative studies	Non-empirical literature (do not contain their own data, e.g., protocol papers, book chapters, comments, editorials). Review protocols. Grey literature.
Language	English	Non-English
Publication date and type	No limitation, search up until 18/12/2024	No full text available (e.g. conference abstracts)

### 2.1 Data extraction and analysis

Data extraction was completed with a data extraction template that collected information on intervention details, study setting and context and main results. Results (both quantitative and qualitative) are reported descriptively, in line with scoping review methodology, with a high-level overview of the main findings. Results are organised according to the technology modality of the interventions (e.g., smartphone applications, web-based programs, etc.) to facilitate a clearer understanding of how different digital formats are being used to support people who use methamphetamine. This approach enables direct comparisons across studies employing similar technological platforms, highlights trends in intervention design and delivery, and allows for more targeted identification of strengths, limitations, and gaps associated with each modality. Given the diversity of digital health technologies and their varying degrees of interactivity, scalability, and clinical integration, this structure provides a practical and meaningful framework for synthesizing and interpreting the evidence base.

### 2.2 Data sources and searches

The search strategy was initially drafted for EMBASE in the Ovid platform (**Table 2**) and was adapted for the remaining databases (Pubmed, PsychINFO, Scopus). The search strategy was iteratively developed and refined by author’s input and a librarian at the University of New South Wales. Searches were adapted according to each database. The search was restricted to studies in English. No start date limit was applied; the search included all literature published up to 18/12/2024.

**Table 2:** Search strategy (EMBASE).

Search Terms	
1.	methamphetamine/
2.	metamfetamine.mp.
3.	amphetamine/
4.	(methamphetamine or amphetamine or crystal meth).mp. [mp=title, abstract, heading word, drug trade name, original title, device manufacturer, drug manufacturer, device trade name, keyword heading word, floating subheading word, candidate term word]
5.	1 or 2 or 3 or 4
6.	“substance use”/
7.	substance abuse/
8.	drug dependence/or amphetamine dependence/or methamphetamine dependence/
9.	Drug Addiction.mp.
10.	6 or 7 or 8 or 9
11.	internet/or web-based intervention/or exp mobile phone/or exp digital health/
12.	(SMS or “Short Message Service” or Texting or “Text Message” or “SMS Reminder” or “Text Message Reminder” or “Digital Health” or “eHealth” or “mHealth” or “Mobile App” or “Mobile Application” or “Mobile Health” or “Wearable Technology” or “smartphone app” or “internet-based” or “web-based” or “virtual reality”).mp. [mp=title, abstract, heading word, drug trade name, original title, device manufacturer, drug manufacturer, device trade name, keyword heading word, floating subheading word, candidate term word]
13.	10 or 11
14.	5 and 10 and 12

## 3 Results

**Figure 1:**
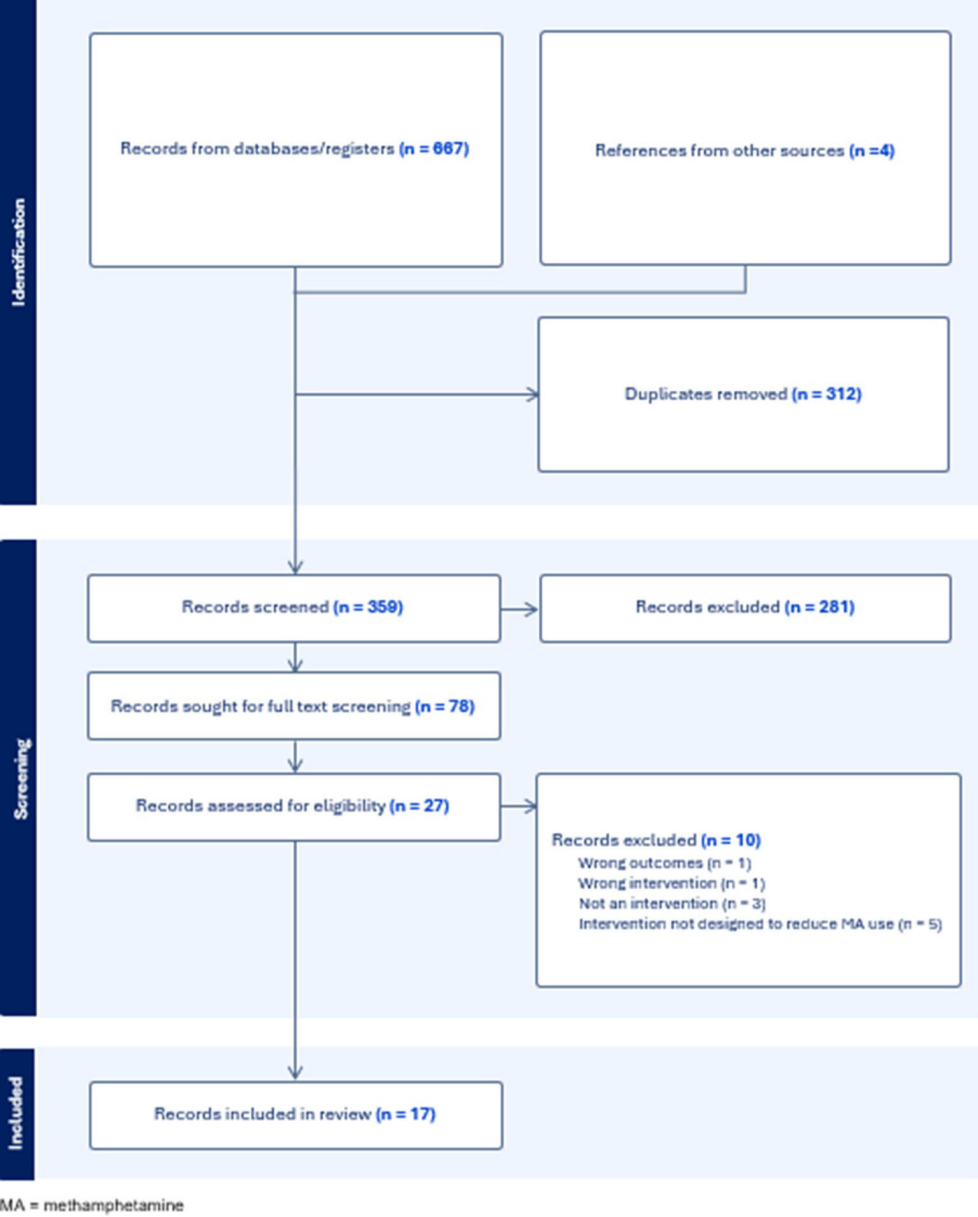
PRISMA breakdown of search results.

Four phases of review were conducted: identification, screening, eligibility assessment and final synthesis (see **Figure 1**). A total of 667 records were retrieved through database searching, with an additional four obtained through citation searching. All entries were exported into Covidence (26) where 312 duplicates were removed. The remaining 359 were screened based on Title/Abstract, of which 281 were excluded. Full text review of 78 records was undertaken by the first author, of which 51 were excluded prior to full eligibility assessment due to failure to meet inclusion criteria upon preliminary inspection. The remaining 27 were randomly assigned and independently assessed for eligibility by two co-authors. Disagreements were discussed between the reviewers and resolved. Reasons for exclusion are summarised in **Figure 1**.

A total of 17 records were subsequently included in the final synthesis, with an aggregate sample of 1269 participants [excluding 102 historical-control participants from the 2018 study conducted by Reback and colleagues (27), who were not prospectively recruited for the intervention], aged 18 and over, across 6 countries. Thirteen studies reported the gender of 1217 participants, with only 180 (14.8%) being women. Results are summarised in **Supplementary Table 2**. Within the 17 records (describing 15 studies), 13 unique interventions meeting the scoping review criteria were identified. Of these, 2 (15.4%) were web-based programs, 2 (15.4%) were text-messaging interventions, 6 (46.2%) were smartphone apps, 1 (7.7%) used VR, and 2 (15.4%) were chatbots or virtual agents. Wearable technology was not used as an intervention in any of the included studies. CBT approaches underpinned 8 of the 13 (61.5%) interventions. Just over half (8/15, 53.3%) of the interventions were subjected to randomised controlled trial (RCT) designs. Across studies, most participants (772/1269, 60.8%) met study inclusion criteria for “methamphetamine use” (timeframes varying from the past month up to the past 12 months), with the remainder (497/1269, 39.2%) meeting diagnostic criteria (DSM-IV or DSM-5) for methamphetamine dependence or use disorder.

### 3.1 Web-based programs

Two RCTs to date have examined web-based programs. Tait and colleagues (28, 29) reported 3- and 6-month outcomes from a single RCT involving 160 community members (31.1% women) who self-reported amphetamine-type-stimulant (including methamphetamine) use in the past 3 months. The intervention (‘*Breakingtheice*’) was a fully automated, self-guided 3-module program based on CBT and MI, designed to reduce stimulant use. While both intervention and waitlist control groups demonstrated statistically significant reductions in stimulant use over 6 months, no between-group differences were observed at either timepoint. High attrition (51% overall dropout rate at 6-months) and limited engagement (37% of the intervention group did not complete any modules), likely constrained intervention effects. Nonetheless, the intervention group showed a significant increase in help-seeking behaviour at 6 months. Additionally, significant reductions in functional impairment (i.e. days out of role) were found in the intervention group at both follow-ups.

The second RCT (30) examined outcomes of 48 outpatients (31.3% women) diagnosed with a substance use disorder, of whom 50% (24 out of 48 participants) identified methamphetamine as their primary drug. Participants were randomised to a digital adaptation of the Serigaya Methamphetamine Relapse Prevention Program (*e-SMARPP*), originally based on the Matrix Model for stimulant users (31), alongside treatment as usual (TAU; medication and face-to-face individual or group psychosocial treatments). The intervention consisted of six web-based relapse prevention sessions delivered over an 8-week period, in addition to a self-monitoring tool, service information and resources, and tailored therapist feedback. While no statistically significant group differences were found in abstinence duration or relapse risk across all drug types, the intervention group showed a moderate effect size (*d* = 0.42, p= 0.16) with a longer mean self-reported abstinence duration (48.8 *vs*. 41.2 days out of 56). Dropout rates were relatively low, with 74% completing all sessions and a 26% dropout rate in the intervention group at 8-month follow-up. However, engagement remained a challenge, with 56.5% completing all six sessions. In a secondary analysis of their pilot RCT, Takano et al. (32) explored whether specific subgroups derived greater benefit from *e-SMARPP*, despite original trial showing no significant overall effect. The most notable finding emerged among participants with short-term outpatient treatment histories (<3 years). Within this subgroup, those in the intervention group achieved 100% complete abstinence during the 8-week intervention period, compared to 58.3% in the control group, with a large effect size (d = 0.96) for abstinence duration. Participants who identified methamphetamine as their primary drug also showed a larger effect size for abstinence duration (d = 0.71) than the full sample (d = 0.42). While 84.6% of those in the intervention group who used methamphetamine achieved complete abstinence (i.e. abstinence on all days during the 8-week intervention period) compared to 54.5% in the control group, this difference did not reach statistical significance. The authors concluded that while the program showed positive effects in certain subgroups, definitive conclusions could not be drawn as there were no interaction effects and the sample size was small, with findings highlighting the need for consideration of individual characteristics when designing future RCTs, or when tailoring treatment approaches for people with diverse backgrounds.

### 3.2 Text-messaging

In their randomized crossover pilot study, Keoleian et al. (33) evaluated the feasibility and preliminary impact of a CBT-based text messaging intervention as an adjunct to outpatient group therapy among five individuals (20% women) with methamphetamine dependence. Participants experienced both an active phase—during which they received four daily CBT-informed text messages plus optional on-demand messages during cravings. These messages included personalized content informed by each participant’s background (e.g. reasons for quitting). In contrast, the placebo period consisted of similarly timed messages containing neutral, non-therapeutic content (e.g., “Paris is the capital of France”). This design allowed assessment of differences in perceived usefulness, engagement, and self-reported substance use between therapeutic and non-therapeutic messaging conditions. The study demonstrated high engagement, with 79% of scheduled assessments completed, rising to 92% when excluding one participant who discontinued due to arrest. Participants were significantly more likely to rate CBT-based messages as “very” or “extremely” useful than placebo messages. While personalised messages were rated more useful than generic ones, this difference was not statistically significant. Self-reported methamphetamine use was slightly lower in the active period (1 reported day of use) compared to the placebo period (2 days), though the study was not powered to detect significance.

Reback et al. (34) conducted an RCT evaluating text-messaging interventions for reducing methamphetamine use and HIV-related sexual risk behaviours among men who have sex with men not currently in treatment. A total of 286 men were randomized to one of three conditions: peer-delivered messaging with automation (TXT-PHE), automated-only messaging (TXT-Auto), or weekly self-monitoring assessments only (AO). Across all groups, significant reductions were observed in self-reported days of methamphetamine use, episodes of sex while on methamphetamine, and condomless anal intercourse with casual partners (men). The TXT-Auto group uniquely demonstrated greater reductions than controls in condomless anal intercourse with anonymous partners and in sex while on methamphetamine, with marginal statistical significance (*p* ≤ 0.10). Peer-delivered messaging did not confer any additional benefits over automated messaging. Improvements were observed for multiple outcomes—including methamphetamine use and HIV sexual risk behaviours—in both TXT-PHE and TXT-Auto groups through the 9-month follow-up, suggesting intervention effects beyond the 8-week active phase. Dropout rates were relatively low across the different timepoints, ranging from 16% at 8-weeks through to 7.3% at 9-month follow-up, supporting text-messaging as an engaging modality over the longer-term.

### 3.3 Smartphone apps

Two RCTs evaluated smartphone apps for methamphetamine use. The first (35) evaluated the preliminary efficacy of “WonderLab Harbor”, an app designed to treat methamphetamine use disorder (MAUD) in a community-based rehabilitation setting. The intervention integrated multiple evidence-based components – including CBT, CM, approach bias modification – designed to reduce craving and enhance cognitive functioning. A total of 100 participants (14% women) diagnosed with MAUD were randomised to the intervention (n = 52) + TAU (weekly telephone counselling sessions from mental health professionals, average duration 11 minutes) *vs*. TAU (n = 48). At 8-weeks, the intervention group demonstrated statistically significant reductions in cue-induced craving and improvements in cognitive function. No significant pre-post changes were observed for either group in terms of self-reported depression or anxiety. App usage and retention were tracked for 40 weeks. Attrition (i.e. stopping use of app altogether) was 11.5% at 8 weeks (increasing to 38.5% at 24-weeks and 55.8% at 40-weeks). High initial engagement was observed with 4.87 (60.9%) CBT sessions (out of a possible 8 sessions) completed at 8-weeks. This was followed by a plateau observed after week 20, reflecting diminishing engagement with structured therapy content over time. Further, engagement was strongest for reward-based and interactive components (CM, approach bias modification and cognitive training) as reflected by higher cumulative usage data over 40 weeks for these features relative to CBT sessions.

In a later RCT, Siefried et al. (15) evaluated the “S-check” smartphone app in a 28-day wait-list-controlled trial targeting community members who self-reported methamphetamine use at least once in the past month. The study aimed to assess whether immediate app access could increase professional help-seeking and enhance motivation to reduce methamphetamine use. A total of 259 participants (25.1% women) were randomised, of which 74% met criteria for high-risk methamphetamine use. Automated feedback was provided based on self-assessment of risks and harms of methamphetamine use across 6 domains (methamphetamine use, sexual health, social health, psychological wellbeing, physical and cognitive health), and tracking functionality, feedback and resources were provided in-app. Primary outcomes showed that a significantly higher proportion of participants in the intervention group reported seeking professional help by day 28 compared to controls (45.5% *vs*. 23.5%). However, no between-group differences were observed in motivation to change or intention to seek help. Ancillary analysis (using imputed data) found that among intervention participants retained to day 28 (n = 33), each additional 10 minutes of app use was associated with a 0.4-day reduction in methamphetamine use over the 28-day period. Overall attrition was high (32.4% provided data at day 28), highlighting a significant challenge in sustaining user engagement. The authors concluded that S-Check is a feasible, low-threshold intervention to reduce barriers to help-seeking for those who use methamphetamine.

Several uncontrolled studies have explored acceptability and feasibility of smartphone apps for people who use methamphetamine. Rabiei et al. (36) evaluated a relapse-prevention app for MAUD, designed to deliver personalised support through self-monitoring, motivational messages and educational content based on Marlatt’s cognitive-behavioural relapse prevention model. The app was tested by 5 experts in addiction psychology, and 5 participants with MAUD (gender not reported) who were receiving care in a community-based addiction rehabilitation centre. High satisfaction was reported, with an average satisfaction of 91.1% across three evaluation domains (usability, content quality, and learning objectives). The most highly rated features by these participants included the accuracy and relevancy of information, the clarity and usefulness of feedback mechanisms, and the ease of navigation and speed of interface. The authors concluded that these findings supported the feasibility and acceptability of the app for individuals in treatment with MAUD, recommending further evaluation in a larger clinical population.

Muhlner et al. (37) evaluated the feasibility and clinical utility of the ‘Affect Digital Therapeutic Program’, a smartphone-based intervention for MAUD, aiming to reduce methamphetamine use and support recovery. The program integrates therapeutic tasks, CM incentives (e.g. rewards for methamphetamine-negative saliva tests and task completion), remote biological drug testing, in-app group and individual CBT, and clinical oversight from health professionals. A total of 49 community members (49.0% women) with MAUD enrolled in the study; most (93.9%) reported methamphetamine use within the past 30 days and none were in formal treatment. The intervention was found to be feasible and acceptable, with 55% completing the full 8-week program. The authors noted that financial incentives through CM played a key role in retention. Further, 12 participants voluntarily continued to use the app post-trial without CM incentives, supporting acceptability and user satisfaction. From week 1 to week 8, participants in the *Affect* program were significantly more likely to provide methamphetamine-negative saliva drug tests compared to baseline, and self-reported abstinence increased over time. Craving scores did not change significantly. At one-month follow-up, 56% of the 16 participants who responded reported no methamphetamine use in the previous 30 days. Participants also reported positive wellbeing outcomes, with 39% rating their improvement in the drug use domain as “10” (much better) and 52% doing so for overall health. The authors concluded that these findings support the program’s feasibility and potential clinical benefit for individuals with MAUD, especially among those who remained engaged.

Hallgren et al. (2023) piloted an adapted version of the “DynamiCare Health” program as a 12-week, fully remote intervention targeting MAUD, with the aim of reducing methamphetamine use or abstaining from use. The program included CM, app-prompted remote saliva-based drug testing, 35 self-paced CBT modules and weekly motivational video calls with a CM guide who had lived experience in recovery. The single-arm study aimed to assess feasibility, engagement and usability of the intervention. Of the 28 (17.9% women) community-based outpatients enrolled, 86% met criteria for severe MAUD and 93% reported methamphetamine use in the past 30 days. Of the 28 participants, 15 (54%) completed the onboarding phase and accessed the program. Engagement varied: 35% of prompted drug tests were completed on average; participants completed an average of 11.5 out of 35 (32.9%) CBT modules and 5.6 out of 12 (47.7%) CM guide calls. Moderate to strong agreement ratings were obtained with respect to ease of use, satisfaction, usefulness for health and wellbeing, and comfort/confidence in using the program to communicate with the CM guide. Engagement with saliva-based drug testing was variable, with low overall rates of verified methamphetamine and amphetamine abstinence (12% out of all tests that were prompted; 31% out of all tests completed). These rates may have reflected a variety of factors including participants potentially skipping tests following recent substance use (particularly as no rewards were offered for completing tests); practical barriers (e.g. forgetting test kits); or the relatively short intervention period (disallowing enough time for sustained changes to be made to support abstinence) (38). The authors suggested that future interventions could improve engagement by offering incentives for test completion regardless of results and incorporating additional support strategies. Preliminary clinical outcomes from baseline to 12 weeks for the 39% (11/28) of participants who received the intervention and completed measures at both timepoints included i) significant reductions in DSM-5 MAUD severity, ii) no significant changes in self-reported depression scores, methamphetamine abstinence self-efficacy, or social support. The authors concluded that a fully remote CM-based intervention for methamphetamine use is feasible for some individuals, but improvements are needed to increase uptake and impact. Suggested enhancements included higher entry-point rewards, incentives for all test completions, streamlined onboarding, and stronger human connection and motivational support, especially early in the program.

Reback et al. (27) conducted a pilot RCT with a quasi-experimental historical control group to assess the feasibility and preliminary efficacy of an ecological momentary assessment (EMA)-based mobile health intervention for methamphetamine-using men who have sex with men. A total of 52 participants were randomised to one of two intervention arms: EMA-only (self-monitoring of methamphetamine use via mobile prompts) or EMA + Counsellor (EMA plus weekly counselling sessions delivered by trained staff. A non-randomized historical control group (n=102) provided a comparison for treatment effects. The intervention aimed to reduce methamphetamine use and HIV-related sexual risk behaviours. At baseline, 97% reported methamphetamine use in the past 30 days. The study found no significant differences between either intervention group and the historical control in self-reported methamphetamine use (days used in the past 30 days), nor urine drug screen results. A significant reduction in episodes of condomless anal intercourse with non-primary partners was observed only in the EMA + Counsellor group compared to controls; no such effect was found in the EMA-only group, suggesting the addition of counsellor support enhanced the intervention’s effectiveness in reducing sexual risk. Treatment retention rates did not differ significantly between groups, with higher dropout rates in the EMA + counselling group (33%) possibly due to additional time commitments. EMA engagement was strong across both arms, with participants completing up to five EMA prompts per day, however the authors noted that financial incentives may have positively influenced adherence.

### 3.4 Chatbots and virtual agents

Three studies to date have examined chatbots and virtual agents. One RCT (39) evaluated the feasibility and preliminary effectiveness of a chatbot-assisted therapy (CAT) program for individuals with MAUD over a 6-month period. The intervention, delivered via smartphone, integrated an 8-session mindfulness-based relapse prevention training program, daily interactive support (automated reminders and encouraging messages to reinforce engagement), mood and symptom tracking with personalised feedback via chatbot, guided meditation sessions, psychoeducational resources and a support service information. Participants (n = 99; 18.2% women) had a diagnosis of MAUD. Compared to controls, participants in the CAT group submitted significantly fewer methamphetamine-positive urine drug screens (19.5% *vs*. 29.6%). Treatment retention was higher in the CAT group (66% *vs*. 51%), although this different was not statistically significant. The overall dropout rate was 41%, with the most common reasons being loss of contact or incarceration. High satisfaction was reported: 84% of participants said they were satisfied with the CAT program, and 95% felt it helped them address their substance use issues. However, satisfaction levels were not collected from those who dropped out of the intervention, and no qualitative feedback was obtained. The authors concluded that CAT is a promising and feasible approach for supporting recovery from MAUD. They noted that future adaptations should consider tailoring interventions to subgroups with more severe methamphetamine use, polysubstance use, or lower readiness to change, who may face greater engagement barriers.

Two single-arm pilot studies evaluated “Echo-App”; a tablet-based virtual digital psychotherapist app designed for people with MAUD. One (40) focused on feasibility and preliminary efficacy (abstinence and treatment motivation, craving reduction); the other (41) examined acceptability and usability (user satisfaction and preferred features). The intervention was a one-time therapy session (30-45min), delivered to inpatients diagnosed with MAUD in a rehabilitation centre, encompassing 4 core modules (self-assessment; treatment via 10 topics drawing from CBT, motivational enhancement therapy and mindfulness-based relapse prevention approaches; homework; feedback) and interactions with a virtual therapist avatar (Virtual Agent; VA). In the first study (40), 47 participants (gender not reported) reported significant increases in abstinence motivation and treatment motivation, along with a significant reduction in methamphetamine craving following the intervention. Significant improvements in perceived importance of abstaining and confidence in their ability to abstain from methamphetamine were also reported post-intervention, suggesting the intervention not only increased motivation but also supported key cognitive shifts linked to behaviour change. In the second study (41), the acceptability of different psychotherapy modalities – including traditional face-to-face (FTF) therapy, online video psychotherapy, VA-led psychotherapy (as per Echo-App) and email psychotherapy - was assessed with 59 men. Participants rated FTF psychotherapy as significantly more acceptable than email-based therapy, while the preference for FTF over VA-led psychotherapy was slightly higher but not statistically significant. Despite this, participants expressed willingness to use VA-led psychotherapy for a variety of mental health concerns, most commonly sleep problems (46.9%), depression (40.8%) and substance use (38.8%). Qualitative data identified features facilitating or acting as barriers to use of VA-led psychotherapy. Facilitators included detailed and thought-provoking treatment content, a vivid virtual therapist, flexible scheduling, novelty, and emotional approachability. Barriers included a preference for human interactions, mistrust in efficacy and VA’s inability to understand human emotions.

### 3.5 Virtual reality

Two studies (42, 43) conducted within detoxification and rehabilitation contexts examined the efficacy of a virtual reality counterconditioning procedure (VRCP) to reduce methamphetamine cue-induced craving. Participants (all men) in both studies (inpatients diagnosed with MAUD) watched VR videos depicting methamphetamine-related cues followed by aversive consequences (e.g., arrest, health complications). The first study (42) of 61 participants found that VRCP significantly reduced craving scores compared to a wait-list control group (p < 0.001). However, the VRCP did not produce a statistically significant group difference in intention to use methamphetamine, although both groups showed a reduction over time. Additionally, the intervention group exhibited significantly greater reductions in heart rate variability indices relative to controls at follow-up, indicating decreased physiological reactivity to drug-related cues. The second study (43) found that prior to VRCP, participants (N = 29) exhibited significantly higher self-reported craving scores and cue-induced gamma-band electroencephalogram activity compared to healthy controls when exposed to methamphetamine-related cues in the VR environment. Following the VRCP intervention, methamphetamine-dependent participants demonstrated significant reductions in craving, liking, intention to use and gamma-band activity. The authors concluded that the intervention effectively reduced both subjective craving and neural reactivity to drug-related cues. Taken together, findings from both studies support VRCP’s potential to attenuate craving responses in men with methamphetamine dependence, but its impact on behavioural intentions to use methamphetamine may vary.

## 4 Discussion

DHIs offer innovative solutions that have potential to extend the reach of treatment and support for people who use methamphetamine. With no regulatory-approved medications for MAUD (44), and psychotherapy providing only modest and short-lived benefits (13, 45), further research into digital health approaches is essential. The number of DHIs in this area is growing, however their focus to date has predominantly been on other substances (e.g., alcohol). This scoping review identified 13 unique DHIs using a range of personal technologies seeking to reduce methamphetamine use and/or its impacts. Results were grouped by technology modality to allow for clearer comparison across intervention types and help identify modality-specific trends, gaps, and strengths. This structure also offers practical guidance for designing future interventions tailored to specific digital platforms. Study findings highlight emerging evidence supporting the feasibility, acceptability and potential efficacy of DHIs in addressing methamphetamine use and related harms, while also identifying limitations and gaps that warrant further research. Key themes and insights are now discussed.

### 4.1 DHIs show promise for people who use methamphetamine

The included studies demonstrated that various DHIs are feasible, can engage people who use methamphetamine and, in some cases, reduce use. Web-based interventions (*Breakingtheice* and *e-SMARPP*) showed mixed findings (28–30). While neither led to significant differences in abstinence rates compared to controls, subgroup analyses suggested greater abstinence benefits for individuals with shorter outpatient treatment histories (32). Further, while these interventions did not appear to directly reduce methamphetamine use, they may be useful for promoting help-seeking behaviour, reducing functional impairment and supporting abstinence maintenance. Text-messaging interventions (33, 34) were demonstrated to be feasible, with high engagement rates and reductions in sexual risk behaviour. However, their impact on methamphetamine use remains unclear. Positively, reductions in use were observed across different text messaging groups and this was maintained over time (34), suggesting that behaviour change can be maintained after relatively brief, tech-based interventions. Interestingly, self-monitoring alone was enough to reduce both methamphetamine use and high-risk behaviours (34), suggesting that regular reflection in and of itself can support behaviour change. Given this, research studies adopting self-monitoring as a control group should consider this as an active control, which may have obscured intervention impacts in previous studies adopting this as a component. Smartphone apps such as *WonderLab Harbor* and *S-Check* demonstrated significant reductions in craving and improvements in cognitive function (35) and increased help-seeking behaviour (15). Chatbot and virtual agent interventions are promising new technology modalities, showing significant reductions in methamphetamine-positive urine tests (39) and improvements in abstinence motivation and craving (40, 41), although some participants preferred human-led therapy. Similarly, VR counterconditioning effectively reduced cue-induced craving (42, 43), though its impact on methamphetamine use behaviour was mixed. However, these novel studies were conducted in samples heavily skewed towards men, limiting generalisability.

### 4.2 Moving beyond substance use and abstinence outcomes

Although outside the scope of this review, we identified DHIs for people that use methamphetamine that, while not specifically designed to reduce use, positively influenced related risk factors. A pilot RCT of a mobile computerized cognitive addiction therapy (CCAT) app reported significant improvements in cognitive function and impulse control tasks in patients with MAUD relative to those receiving treatment-as-usual (46). Cognitive impairments - particularly in executive functions and memory – are hypothesised to hinder treatment and raise relapse risk in people who use methamphetamine (47); targeting these deficits through cognitive rehabilitation may enhance long-term outcomes. Qi et al. (48) tested a VR competitive cycling exercise in methamphetamine-dependent men, finding that a single 15-minute session enhanced cognitive performance, mood, and brain network efficiency, suggesting potential for cognitive and emotional regulation improvements. Other studies suggest that text-based interventions are feasible, engaging and have potential to change methamphetamine use behaviour. For example, several studies have examined text-based interventions aimed at improving antiretroviral therapy (ART) adherence in HIV-positive people who use methamphetamine, with incidental findings on methamphetamine use. Moore et al. (49) demonstrated that personalised text messaging was a feasible and engaging way to monitor ART adherence and methamphetamine use. In their later RCT (50), a trend towards reduced methamphetamine use was reported in the intervention group. A third study (51) examined a smartphone app intervention (“APP+”) to improve ART adherence in men who have sex with men who use stimulants (including methamphetamine). While ART adherence was the primary aim, this intervention included harm reduction strategies and information on stimulant use, and messages aimed at reducing use. At 4-month follow-up, significantly fewer men in the intervention group reported recent stimulant use compared to controls (p = 0.02), with this trend continuing but weakening (p = 0.05) at the 6-month follow-up. Further research is needed to explore how text-based interventions can be optimised, particularly in terms of incorporating more direct strategies for methamphetamine use reduction. Finally, while reducing methamphetamine use may be a key goal, promoting help-seeking is important, given delays in treatment initiation (10) and low uptake among those with substance use disorders (52). DHIs may help facilitate earlier treatment seeking, particularly for treatment naïve individuals, as evidenced in two studies in this scoping review (15, 29).

### 4.3 Engagement and retention remain major challenges

Despite promising initial engagement, declines were observed across DHIs over time. Further, dropout rates were high across multiple interventions and timeframes, ranging from 7.3% to 67.7%. Web-based and app-based programs showed the greatest attrition, with up to 67.6% of participants disengaging within weeks or months. As noted previously (19), low levels of engagement might have masked the true treatment effect in prior studies (e.g., (29, 30). Consistent use of DHIs may play a meaningful role in supporting people to achieve their treatment goals. Indeed, for substance use disorders more broadly, greater engagement (i.e. number of modules completed over time) positively correlated with the probability of abstinence in the last four weeks of treatment among those who completed a 12-week DHI treatment program (53). In a large-scale study evaluating retention and engagement across eight remote digital health studies for a range of conditions involving over 100,000 participants (54), high attrition rates were reported (median 5.5 days), with more than half of participants discontinuing within the first week and low sustained participation (i.e. most participants performed in-app tasks for only two days within the first 12 weeks). Notably, those who had the clinical condition of interest had improved engagement, suggesting that those experiencing relevant symptoms or challenges may be more motivated to engage with digital tools, highlighting the need for interventions that are designed to explicitly target the condition of interest. Given the relative paucity of DHIs designed specifically for people who use methamphetamine, there is a clear need for development and evaluation of methamphetamine-specific tools.

It is important to note that high attrition patterns are not unique to DHIs. Average dropout rates for in-person psychological treatments for SUDs are 30%, though they vary significantly by population, substance, and treatment focus; notably, studies targeting methamphetamine have reported rates as high as 53.5% (55). This suggests that waning participation may reflect broader patterns in the varying motivation and treatment engagement among people who use methamphetamine or other substances. Indeed, psychosocial approaches are frequently accessed in a non-linear manner, with individuals dipping in and out of care depending on perceived need, life circumstances, or readiness for change. Rather than viewing disengagement as treatment failure, it may be more constructive to design DHIs that accommodate these fluctuating patterns, enabling flexible re-engagement and acknowledging that substance use disorders are dynamic.

Findings from some studies (35, 37) in the current scoping review suggested that DHIs incorporating CM or reward-based engagement tended to have higher retention rates, therefore financial incentives may improve adherence in this population. Emphasising personalised benefits (e.g. craving reduction, cognitive improvements, potential for reduced methamphetamine use subject to ongoing engagement) may also serve as an effective engagement strategy.

### 4.4 A case for personalisation in DHIs – “one size doesn’t fit all”

Findings from this review highlight substantial variability in intervention effectiveness, engagement, and outcomes across different populations and intervention types. This underscores the importance of designing DHIs that are not only personalised, but also equity- informed.

For example, in the ‘*Breakingtheice*’ program (28, 29) over half of participants dropped out by six months, and more than one-third did not complete any modules. These figures raise concerns about the accessibility and appropriateness of self-guided web-based programs, particularly for individuals with limited digital literacy, unstable internet access, or co-occurring psychosocial challenges. Some may benefit from more structured support, while others may only require brief engagement if their needs are met earlier - highlighting the need for flexible and responsive delivery models.

In the ‘*e-SMARPP*’ trial (30), individuals with shorter treatment histories or whose primary substance was methamphetamine (rather than multiple substances) showed greater positive outcomes. This suggests that treatment history and substance use profiles influence who benefits most, pointing to the need to tailor interventions to individual circumstances.

Likewise, chatbot-assisted therapy (39) was less effective for individuals with high-severity use, polysubstance dependence or low readiness to change. The authors also noted that participants may have been more comfortable with digital technologies, potentially excluding those who prefer human contact or are sceptical of automation.

Taken together, these findings support the development of personalised, adaptable DHIs that respond to individual readiness, preferences, and social contexts, rather than a one-size-fits-all approach. Participatory co-design, involving people with lived/living experience from a range of backgrounds, from the earliest design phases through to evaluation, is likely to ensure that DHIs are relevant, culturally appropriate, engaging, and capable of supporting meaningful behaviour change (56). Finally, applying an equity lens to design and implementation will help ensure DHIs reduce, rather than exacerbate, disparities in access, engagement, and outcomes (57).

### 4.5 Limitations and study constraints

A limitation of this review was the lack of inclusion of grey literature, which may have excluded relevant insights from government reports, clinical guidelines, and unpublished studies. We excluded grey literature to focus on peer-reviewed studies that met minimum standards of methodological quality and reporting. While this improves the reliability of findings, it may contribute to publication bias and omit potentially informative real-world program evaluations and implementation data from non-peer-reviewed sources. In line with scoping review methodology, a formal critical appraisal of study quality was not conducted. However, key methodological features and limitations are now briefly overviewed to contextualise the findings.

While digital interventions show promise, the studies in this review exhibited varying methodological rigour. There was substantial heterogeneity across study designs, populations, outcome measures and intervention components, limiting comparability. Nearly half of the studies were small-scale pilot trials, single-arm feasibility studies, or lacked control groups, constraining conclusions about causal effects. Most studies also did not assess long-term outcomes, making it unclear whether benefits were sustained. This highlights the need for larger, well-powered RCTs with extended follow-up periods to assess durability of effects on methamphetamine use and relapse prevention. Sample sizes ranged from as few as five to 286 participants – with smaller studies limiting generalisability and statistical power. While some studies included participants with a formal diagnosis of MAUD, others relied on self-reported methamphetamine use without diagnostic confirmation. Outcome measures also varied, with some relying on biologically verified data (e.g. urine or saliva drug tests), while others used only self-report. Follow-up periods were inconsistent, and attrition was frequently high - potentially obscuring true efficacy and highlighting the need to improve engagement and retention in DHIs.

Generalisability is further limited by the contextual specificity of many studies, which often targeted narrowly defined groups – e.g. men or those in inpatient rehabilitation settings. Notably, most studies were heavily skewed towards men, leaving uncertainty about how women may respond to these interventions. This is concerning given that in Australia, women are less likely to seek treatment for MAUD (58), despite comparable rates of frequent methamphetamine use (59). Gender-specific interventions may offer a pathway to close this treatment gap, leveraging DHIs to enhance access and engagement. The World Health Organization (WHO) highlights the gender divide in digital health, strongly supporting the development of gender-specific DHIs and policy frameworks to ensure that digital health technologies effectively meet women’s needs and reduce gender-based disparities in healthcare access, treatment and outcomes (60).

Finally, intervention modalities and theoretical underpinnings were also diverse. Approaches ranged from SMS reminders to comprehensive app-based therapies. While this reflects innovation, it also complicates synthesis and limits generalisability. While such diversity highlights innovation in the field, it also poses challenges for synthesis and limits the generalisability of findings. Most interventions were informed by CBT or MI, yet several studies did not clearly articulate their theoretical foundation, making it difficult to assess intervention fidelity or mechanisms of action. The variability in outcomes assessed - from abstinence to craving to engagement - across inconsistent timepoints further complicates interpretation and makes it difficult to determine which interventions work best for which populations.

### 4.6 Future opportunities

To address these challenges and improve the coherence of future research and clinical application, a stepped care framework offers a promising path forward. Drawing from the mental health field, this approach acknowledges that individuals vary in clinical severity, readiness for change, and support needs - and that interventions should be tailored accordingly. It allows for the development and testing of interventions that range from low-intensity, self-guided tools to more comprehensive, clinician-supported services.

For example, light-touch interventions – such as informational apps or SMS-based check-ins - may be appropriate for individuals early in their change journey or beginning to seek help (61). As treatment needs intensify, more structured interventions such as online CBT programs may be warranted, particularly for those with greater severity of substance use disorder or co-occurring mental health concerns. In these cases, hybrid models that combine digital tools with human support - such as clinician-assisted platforms or telehealth counselling - can offer additional scaffolding. At the highest tier, DHIs can be integrated into comprehensive care models, supporting substance use goals in outpatient or inpatient settings and helping maintain continuity of care for individuals with complex clinical or psychosocial needs.

Embedding digital intervention development within a stepped care model could also encourage more consistent use of outcome measures and population stratification – facilitating a clearer understanding of what works best, when, and for whom. Future research should explore how this framework might guide both the design and evaluation of DHIs for methamphetamine use. Emerging innovations in AI-driven therapy, CM, and tailoring may enhance treatment effectiveness and scalability. Finally, it is important to note that while meta-analyses to date (62–64) collectively report that DHIs for substance use disorders have small to moderate effect sizes, the scalability of DHIs allow small individual changes to aggregate into meaningful public health impacts.

This review highlights several critical knowledge gaps: (1) the lack of large-scale, high-quality RCTs with long-term follow-up to assess durability of effects; (2) underrepresentation of women, limiting generalisability; (3) limited understanding of effective engagement and retention strategies for people who use methamphetamine; (4) minimal exploration of hybrid and stepped care models tailored to this population; (5) lack of consistent, standardised outcome measures to enable comparison across studies; and (6) insufficient investigation of how interventions can be personalised and co-designed to meet the needs of diverse users. Addressing these gaps will be essential to advance the evidence base and inform policy and practice.

## 5 Conclusion

This scoping review highlights a growing body of evidence supporting DHIs as potential tools to help people who use methamphetamine. Engagement and long-term retention remain a challenge for these types of interventions. Future studies should focus on sustained intervention effects, personalization, and integration of co-designed digital health solutions into broader treatment frameworks, with development and testing guided by a stepped care approach.
