# Supplementary material for: Digital health interventions for people who use methamphetamine: a scoping review
Source: Front Psychiatry. 2026 Jan 20;16:1658021. doi: 10.3389/fpsyt.2025.1658021 (PMC12864073; doi:10.3389/fpsyt.2025.1658021)
Supplement: Supplementary file 1 [file Supplementaryfile1.docx]

**Supplementary File 1. Preferred Reporting Items for Systematic reviews and Meta-Analyses extension for Scoping Reviews (PRISMA-ScR) Checklist**

**
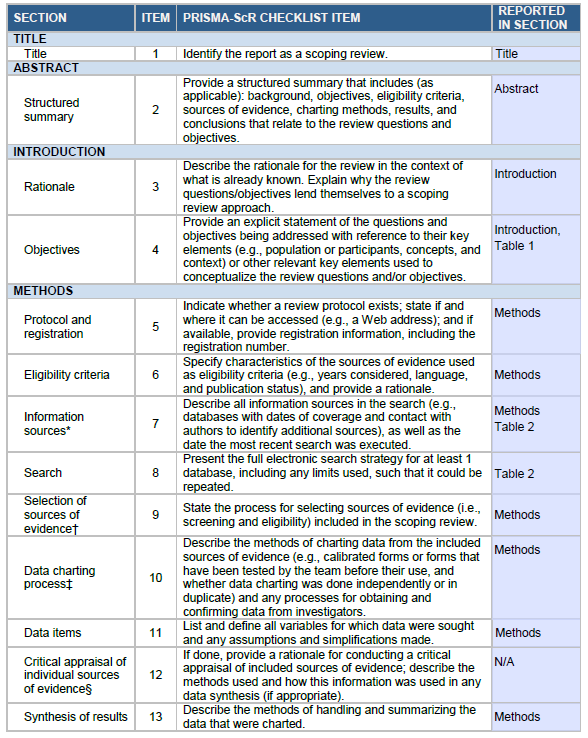
**

**
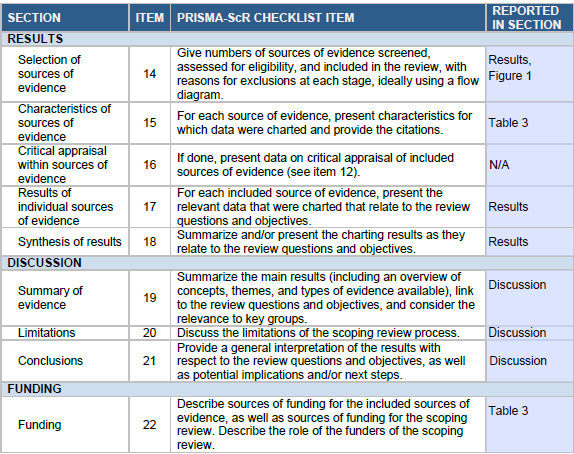
**

**
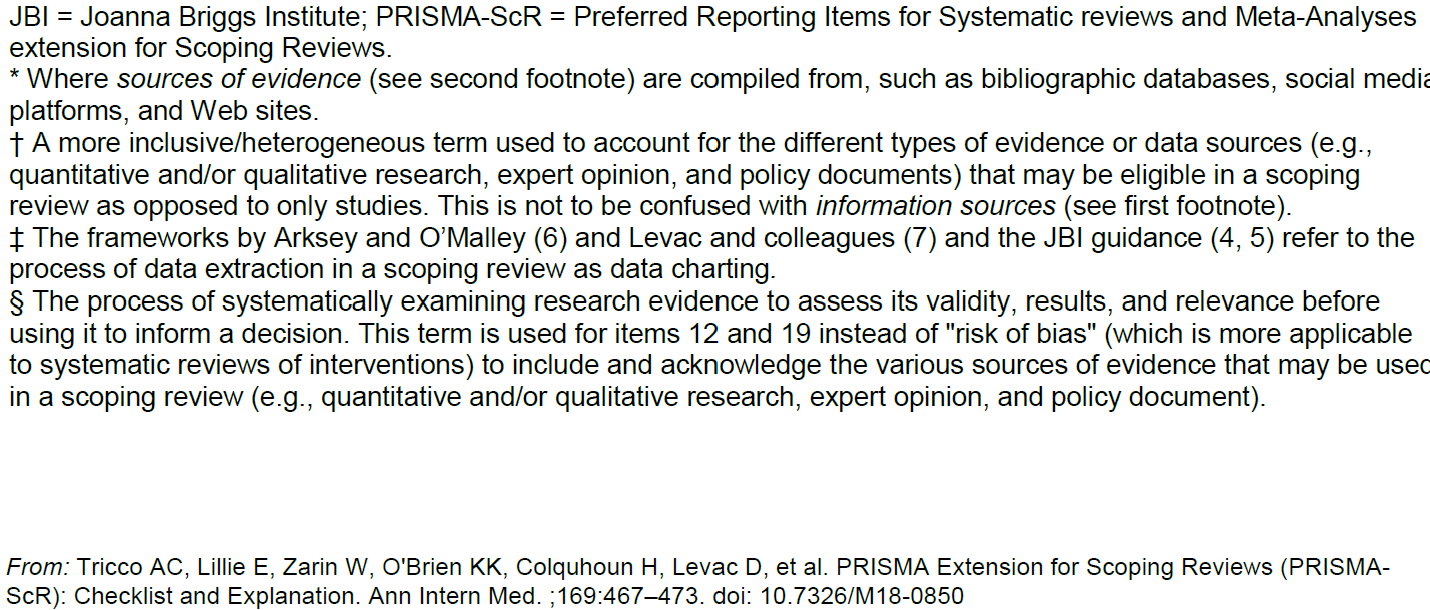
**
