# Supplementary material for: Digital health interventions for people who use methamphetamine: a scoping review
Source: Front Psychiatry. 2026 Jan 20;16:1658021. doi: 10.3389/fpsyt.2025.1658021 (PMC12864073; doi:10.3389/fpsyt.2025.1658021)
Supplement: Supplementary file 2 [file Supplementaryfile2.docx]

**Supplementary File 2. Characteristics of included studies**

| **Author, year** | **Country** | **Funding** | **Digital health intervention (Name; approach; length)** | **Study characteristics and outcomes**  **(Design; sample characteristics and inclusion criteria; key findings)** | | |
| --- | --- | --- | --- | --- | --- | --- |
| **Web-based programs** | | | | | | |
| Tait et al., 2014;    Tait et al., 2015 | Australia | Commonwealth of Australia, Department of Health and Ageing; National Health and Medical Research Council;Curtin University | Breakingtheice; CBT; 3 modules accessed over 6 months | RCT (wait-list control group) | 160 (122 men, 38 women) community members;  (1) Australian resident, (2) aged 18 years or over, (3) report use of ATS (e.g., meth/amphetamine, ecstasy, nonmedical use  of prescription stimulants) in the last three months, (4) access to internet and valid email address | 3-mth outcomes   - Stimulant use: both groups reduced stimulant use; no significant differences between intervention and controls. - Help-seeking: Actual help-seeking increased more in the intervention group (from 0.3 to 0.6), but this was not statistically significant in intention-to-treat analysis. However, per-protocol analysis (those who completed modules) showed a significant increase (OR = 2.90). - Polydrug use: small non-significant reductions favouring intervention group. - Psychological distress: small non-significant improvements favouring intervention group - Days out of role: significant reduction (p=0.01, Cohen’s *d* = 0.29) in work/social impairment for intervention group. - Engagement and attrition: 63% completed at least one module, 48% completed all three modules. 58% completed 3-month follow-up.   6-mth outcomes   - Stimulant use: both groups reduced stimulant use; no significant differences between intervention and controls. - Help-seeking: significant increase in actual help-seeking (p = 0.02, RR = 2.16) and help-seeking intentions (p = 0.005, RR = 1.17) in intervention group. - Days out of role: significant reductions in complete (RR = 0.50) and partial impairment (RR = 0.74). - Psychological distress, quality of life and polydrug use: no significant improvements. - Engagement and attrition: 37% did not engage with the intervention at all, 48% completed all three modules. 47% of intervention participants completed 6-mth follow-up. |
| Takano et al, 2020;  Takano et al., 2022 (re-analysis of 2020 study) | Japan | Pfizer Research Foundation (Japan); Japan Society for the Promotion of Science  Japan Society for the Promotion of Science | Serigaya Methamphetamine Relapse Prevention Program (e-SMARPP) + TAU (medication, face-to-face individual or group psychosocial treatments); CBT (relapse prevention focus), MI, self-monitoring, information resources, tailored feedback from a web-therapist; 6 sessions accessed over 8 weeks | RCT (self-monitoring and information resources control group + TAU) | 48 (33 men, 15 women) outpatients;  (1) diagnosed with substance use disorder (psychoactive substances other than alcohol and tobacco) assessed by DSM-IV or 5 by a psychiatrist, (2) used a primary abused drug in the past year, (3) access to internet via personal computer, smartphone or tablet computer and could exchange email | Primary outcome (self-reported duration of abstinence over 8-week intervention period):   - No statistically significant differences between groups. However, intervention group had a longer mean duration of abstinence from their primary drug (48.8 days vs. 41.2 days out of 56, Cohen’s *d* = 0.42)​.   Secondary outcomes:   - No significant group differences in relapse risk, motivation to change, self-efficacy, money spent on drugs. - Effect sizes were generally small or inconsistent, except for a moderate effect (Cohen’s *d* = -0.54) in favour of the intervention group​ at the 5-month follow-up on money spent on drugs, which was not sustained at 8-month follow-up.   Program completion, feasibility and attrition:   - 74% of participants in the e-SMARPP group completed all relapse prevention sessions. - Self-monitoring completion across all 8 weeks was >80% in both groups. - 26.1% did not complete the e-SMARPP intervention. - 43.5% of e-SMARPP group did not complete 8-month follow-up   Abstinence:   - Participants with short-term outpatient treatment (<3 years) (n = 10 in intervention group, n = 12 controls) showed the strongest benefit from the intervention:   - Longer abstinence duration (mean = 56.0 days vs. 42.3 days; p = 0.04, Cohen’s *d* = 0.96).   - Higher complete abstinence rate (i.e. 56 days abstinent): 100% vs. 58.3%; p = 0.02). - Participants who identified methamphetamine as their primary drug (n = 13 in intervention group, n = 11 controls) reported more days abstinent on average over the 8-week intervention than controls (51.5 days vs. 40 days, Cohen’s d = 0.71), however this result was not statistically significant (p = 0.12). Further, a higher but non-significant proportion of this group remained completely abstinent during the intervention vs controls (84.6% vs. 54.5%, p = 0.11). - Participants with previous face-to-face relapse prevention experience (n = 10 in intervention group, n = 12 controls) 13 in intervention group, n = 11 controls) reported more days abstinent on average over the 8-week intervention than controls (52.5 days vs. 42.9 days, Cohen’s d = 0.54), however this result was not statistically significant (p = 0.19). Further, a higher but non-significant proportion of this group remained completely abstinent during the intervention vs controls (90.0% vs. 69.2%, p = 0.23). - No significant interaction effects between intervention and subgroup variables. |
| **Text-messaging** | | | | | | |
| Keoleian et al., 2013 | USA | National Institutes of Health (NIH); gift from  Emily G. Kahn. | No name; CBT; 23 days (8- days placebo period, 7-day washout period, 8-days active period) | Feasibility study with randomised cross-over design (placebo text message control group, i.e. neutral, non-therapeutic texts) | 5 (4 men, 1 woman) outpatients;  (1) aged 18 years or over, (2) own a cell phone with unlimited texting, (3) experience high levels of craving for methamphetamine, defined as having at least one craving event of 3 or higher on a 0-10 scale in the past two weeks, (4) willing to participate in a 23-day pilot study, involving text message-based assessments and adjunct group CBT | Feasibility and acceptability:   - All participants found the system easy to use - 79% of scheduled text message assessments were successfully collected; this rose to 92% when excluding one participant who dropped out due to arrest. - Most participants (3 out of 5) were satisfied with the number and timing of messages (4/day). - Messages in the active phase were more likely to be rated "very" or "extremely useful" were 6.6 times vs. placebo (OR = 6.6; 95% CI: 2.2–19.4). - Personalized messages were rated as more useful, although this difference was not statistically significant (p = 0.4).   Methamphetamine use and craving:   - Self-reported methamphetamine use was lower in the active period (1 reported use) than in the placebo period (2 reported uses) – result was unable to be statistically tested due to small sample size (n = 5). - Mean daily peak craving scores were 40% higher in the active vs. placebo phase, however the authors explicitly suggested this may reflect increased awareness triggered by CBT-based messages, not increased actual craving. |
| Reback et al, 2019 | USA | National Institute on Drug Abuse; National Institute of Mental Health | Project Tech Support2; Social Support Theory (peer encouragement), Social Cognitive Theory (behavioural coping strategies), Health Belief Model (risk perception and motivation) delivered over 8 weeks  1) TXT-PHE: interactive text conversations with Peer Health Educators + automated text messages + weekly self-monitoring    (2) TXT-Auto: automated text messages + weekly self-monitoring  (3) AO: weekly self-monitoring only (attentional control) | RCT (self-monitoring control group) | 286 (men) community members;  1) Self-identified men who has sex with men, (2) between the ages of 18-65 years, (3) used methamphetamine within the previous three months, reported condomless anal intercourse with a non-primary partner in the previous 6 months, (4) not currently in treatment or seeking methamphetamine abuse treatment, (5) has a personal cell phone with unlimited texting service and the capacity to charge the phone daily, (6) able and willing to provide informed consent and comply with study requirements | Methamphetamine use and risk behaviour:   - All groups reported significant reductions (all *p* ≤ 0.05) in days of methamphetamine use (coef. = -.10), episodes of sex while on methamphetamine (coef. = –0.09), episodes of condomless anal intercourse with casual partners (coef. = –0.06). These reductions were sustained through the 9-month follow-up. - TXT-PHE was not significantly more effective than TXT-Auto on any outcome. - TXT-Auto showed the greatest reductions in (1) condomless anal intercourse with anonymous partners (coef. = -0.05, p ≤ 0.10) and (2) sex while on methamphetamine (coef. = -0.05, p ≤ 0.10).   Retention:   - 8-week follow-up (intervention end): TXT-PHE 81%, TXT-Auto 85%, AO 86% - 3-mth follow-up: TXT-PHE 93%, TXT-Auto 86%, AO 90% - 6-mth follow-up: TXT-PHE 86%, TXT-Auto 87%, AO 86% - 9-month follow-up: TXT-PHE 95%, TXT-Auto 90%, AO 93%   Engagement:   - No significant differences in response rates across groups; overall average response rate to weekly assessments was 63.2% |
| **Smartphone apps** | | | | | | |
| Zhang et al., 2022 | China | Natural Science Foundation of China; Adai Technology (Beijing) Co.,  Ltd., Beijing, China. | WonderLab Harbor; CBT, approach bias modification, cognitive training, CM + TAU (weekly telephone counselling sessions from mental health professionals, average duration 11 minutes; 8 sessions accessed over 8 wks | RCT (TAU control group) | 100 (86 men, 14 women) outpatients;  (1) aged between 18 and 50 years, (2) meets DSM-IV criteria for methamphetamine dependence | Primary outcome (cue-induced methamphetamine craving):   - Significantly reduced craving in intervention group (Wilcoxon effect size = -0.267, p = 0.002).   Additional outcomes (cognitive function, depression, anxiety):   - Significantly improved cognition function in intervention group (Wilcoxon effect size = 0.220, p = 0.041). - No significant changes in depression or or anxiety scores in either group.   Attrition:   - 8-wk: 11.5% of participants stopped using the app by week 8. - 24-week: 38.5% disengaged with app - 40-week: 55.8% disengaged with app   Engagement:   - At 8 weeks, the most-used feature was daily check-ins (avg. 16.17 uses), and the least-used was iCBT sessions (avg. 4.87). - By week 40, participants had used daily check-ins 51.35 times and completed 8.98 iCBT sessions on average, indicating some re-use but lower engagement with therapeutic content. - Engagement was highest for reward-based and interactive components of the app, as shown by cumulative usage data over 40 weeks. On average, participants completed 51.35 daily check-ins and 27.48 points redemptions (contingency management features), compared to 15.73 cognitive training sessions and 14.31 approach bias modification sessions (gamified components). In contrast, usage of structured therapeutic content was lower, with an average of only 8.98 iCBT sessions completed over the same period, despite all sessions being available for repeat viewing. |
| Siefried et al, 2024 | Australia | New South Wales Health Early Intervention and Innovation Fund; National Centre for  Clinical Research on Emerging Drugs (funded by the Australian Department of Health and Aged Care); St  Vincent’s Hospital Sydney Inclusive Health Program. | S-Check; Self-assessment tools, tailored feedback and persuasive messaging, self-monitoring and tracking, help-seeking support; 28 days | RCT (wait-list control group) | 259 (193 men, 65 women) community members;  (1) aged 18 years or over, (2) living in Australia, (3) self-reported methamphetamine use at least once in the month before study enrolment, (4) private access to a smartphone | Primary outcomes (help-seeking, motivation to change methamphetamine use):   - A significantly higher proportion of those in the intervention group sought professional help (45.5% vs. 23.5%, χ² = 4.42, p =0.04) at day 28. - No differences between groups in motivation to change scores.   Secondary outcomes (methamphetamine use, engagement):   - Adjusting for baseline use among intervention participants (n – 33) in a multivariable regression analysis, each 10-minute increase in app use was associated with a 0.4-day reduction in methamphetamine use (β = –0.04, p = 0.02). - Of 259 participants who consented and completed baseline, 84 (32.4%) completed the 28-day survey.   Retention   - 32.4% completed the 28-day follow-up |
| Rabiei et al, 2020 | Iran | No financial support | No name; CBT, Marlatt’s relapse prevention model; N/A | Acceptability evaluation | 5 (gender not reported)  addiction recovery centre inpatients;  (1) diagnosed with MAUD | Feasibility and acceptability:   - Patient participants evaluated the app positively, with particularly high ratings for usability (mean = 4.6/5) and content quality (4.5/5). - Average satisfaction across all evaluated dimensions (usability, content quality, learning objectives) was 91.1% |
| Muhlner et al, 2023, | USA | Affect Therapeutics, Inc | The Affect Digital Therapeutic Program; CBT, CM, CRA, and group/individual counselling (delivered in-app via videoconferencing); 8 wks | Single-arm pilot study | 49 (20 men, 24 women) community members;  (1) aged 18 years or over, (2) able to read and understand English, (3) be an active methamphetamine user who met DSM-5 criteria for moderate- to severe MAUD, (4) affirm an intent to cease meth use, (5) have a smartphone and be capable of using apps, (6) have a mailing address, (7) have medical insurance coverage, (8) reside in California | Retention:   - 27 out of 49 (55.1%) completed the 8-week program.   Participation:   - On average, program completers (vs. non-completers) (1) attended significantly more meetings (35.3 vs. 6.86, p < 0.001), (2) returned significantly more methamphetamine-negative saliva screens (5.19 vs. 0.33, p = 0.004), and (3) achieved the longest abstinence streak (10.9 days vs. 4.5, p = 0.001).   Methamphetamine use:   - The odd ratio for testing meth-negative from week 1-8 vs. baseline was OR = 1.57, p = 0.03. - Self-reported abstinence increased significantly over time (OR = 1.69, p = 0.031).   Craving:   - No significant change in average craving scores from week 1 to week 8 (p = 0.26). |
| Hallgren et al, 2023 | USA | University of Washington Medicine Garvey Institute for Brain  Health Solutions; National Institute on Alcohol Abuse and Alcoholism | DynamiCare Health Program (Smartphone app + telephone contact with CM guide (peer support specialist); Self-monitoring, CBT, CM; 12 wks | Single-arm pilot study | 28 (23 men, 5 women) outpatients;  (1) receiving care from a participating primary care clinics or speciality substance use disorder clinic, (2) self-reported methamphetamine use for ≥5 out of the past 30 days, (3) self-reported goal to reduce or abstain from methamphetamine use, (4) aged 18 years or older, (5) ability to read and communicate in English per self-report | Uptake and retention:   - 54% of enrolled (15 of 28) participants completed the “welcome phase” and commenced the 12-week program. - 39% completed the 12-week follow-up   Engagement:   - On average, participants completed 25% of substance test prompted by the app. - An average of 11.5 out of 35 CBT modules, and 5.6 out of 12 recommended CM guide calls were completed.   Usability:   - All 12 participants who completed a mid-intervention usability questionnaire agreed or strongly agreed with ease-of-use and satisfaction statements.   Supplemental clinical outcomes:   - Significant reduction in MAUD severity from baseline to week 12 (mean 8.73 to 7.00, p = 0.04, Cohen’s *d* = -0.54). - No significant changes in depression symptoms, methamphetamine abstinence self-efficacy or social support. |
| Reback et al, 2018 | USA | National Institute of  Mental Health; Los Angeles County, Department of Public Health, Division of HIV  and STD programs; City of West  Hollywood, Division of Social Services; William T. Grant  Foundation | EMA (EMA, EMA+Counsellor) via smartphone app (add-on component to the “Getting Off” group program); self-monitoring over 8 weeks | Pilot RCT with a quasi-experimental historical control group | 34 (vs. 102 historical controls) (men) outpatients;  (1) identified as gay or bisexual men, (2) used methamphetamine in the past 12 months, (3) seeking treatment for methamphetamine use in the “Getting Off” adult outpatient program, (4) enrolled in the outpatient methamphetamine treatment program within the first week of participation, (5) owned a smartphone or were willing to use a study-provided phone with a data plan | Methamphetamine use:   - No significant differences between the intervention groups and historical controls for self-reported methamphetamine use (days used in past 30 days), or urine drug screen results.   Sexual risk behaviour:   - Significant reduction in number of episodes of condomless anal intercourse with non-primary partners observed only in EMA+Counsellor group relative to historical controls (IRR = 0.02, p ≤ .01).   Feasibility and engagement:   - Participants completed 86.6% of all prompted EMA messages - No significant difference in EMA completion between EMA and EMA+Counsellor groups.   Retention (defined as either attended a group session, or submitted a urine sample) and follow-up survey completion rates:   - No significant between-group differences with respect to the last week of clinic attendance. - 8 week follow-up: 93.8% of EMA-only group and 66.7% of EMA + Counsellor - 12-week follow-up: 81.3% of EMA-only group and 66.7% of EMA + Counsellor |
| **Chatbots and virtual agents** | | | | | | |
| Chun-Hung et al, 2023 | Taiwan | Integrated Drug Addiction Treatment Center of the  Jianan Psychiatric Center; Ministry of Health and Welfare in Taiwan | Chatbot-Assisted Therapy (CAT); Mindfulness Based Relapse Prevention, recovery support and psychoeducation; 8 sessions over 6 mths | RCT (face-to-face Mindfulness Based Relapse Prevention control group) | 99 (81 men, 18 women) outpatients;  (1) diagnosis of MAUD as defined by DSM-5, (2) age between 18 and 65 years, (3) no acute exacerbation of severe mental illness during the initial assessment, such as schizophrenia or bipolar I disorder, (4) willingness to participate in standard outpatient treatment for at least 6 months, (5) an Android phone | Primary outcomes (methamphetamine use, treatment retention):   - CAT group had significantly fewer MA-positive urine samples than controls (19.5% vs. 29.6%, *F* = 9.116, p = 0.003). - While the CAT group had longer average retention (142.5 days vs. 118.1 days) and a higher completion rate (66% vs. 51%) at 6-month follow-up, these differences were not statistically significant relative to controls.   Secondary outcomes (readiness to change, participants satisfaction, app quality):   - CAT group showed significantly greater increases on on ‘contemplation’ subscale of readiness to change measure (F = 5.6, p = 0.012). - Significant correlations were observed between readiness to change and MA-positive urine results (r = -0.330, p = 0.001) and treatment retention (r = 0.401, p <0.001) across the total sample. - The majority (84%) of participants were satisfied with CAT and 85% agreed it helped address addiction. - App quality rated very high by experts using the Mobile App Rating Scale (mean score = 4.47/5), particularly information (4.95) and functionality (4.50). |
| Li et al., 2024  Chen et al., 2023 | China | National Natural Science  Foundation of China; Shanghai Shenkang  Hospital Development Center; Shanghai  Key Laboratory of Psychotic Disorders; Shanghai Rising-star Cultivation Program; Capability Promotion Project for Research-oriented Doctor at  SMHC; Shanghai Municipal Science and  Technology Major Project; Shanghai  Engineering Research Center of Intelligent Addiction Treatment  and Rehabilitation; Shanghai Clinical  Research Center for Mental Health  National Nature Science Foundation; Shanghai Municipal Science and Technology  Major Project; Shanghai Shenkang Hospital Development Center; Shanghai Rising-star  Cultivation Program; Capability Promotion Project for Research-oriented Doctor at SMHC; Brain Science and Brain-Like Intelligence Technology; Shanghai “the Hospital Garden Star” Funding for  training of young medical professionals; Shanghai Clinical Research Center for Mental Health; Shanghai Engineering Research Center of Intelligent Addiction Treatment and Rehabilitation; Shanghai Key  Laboratory of Psychotic Disorders; Lingang Lab | Echo-App chatbot with web-based modules (delivered by tablet); Cognitive bias modification; 1 x 30-45min session | Single arm pilot study  Single arm pilot study | 59 (men) rehabilitation centre inpatients;  (1) diagnosis by a psychiatrist of MAUD as defined by DSM-5, (2) have received at least nine years of formal education, (b) aged 18-55 years, (c) normal or corrected-to-normal vision and audition, and (d) without severe cognitive deficits/impairments  47 (gender not reported) rehabilitation centre inpatients;  (1) diagnosis by a psychiatrist of MAUD as defined by DSM-5, (2) aged 18-55 years, and (3) normal or corrected-to-normal vision and audition | Acceptability:   - Traditional face-to-face (FTF) psychotherapy was rated as significantly more acceptable than email-based therapy (p = 0.042); no difference between FTF therapy and VA-led psychotherapy (p = 0.059). - Top psychological needs participants indicated they were willing to use VA-led psychotherapy for: sleep problems (46.9%), depression (40.8%), substance abuse (38.8%). - Participants were less willing to use VA-led therapy for sexual distress and relationship issues (each under 20%).   Usability:   - Positive aspects facilitating use of VA-led psychotherapy: detailed and relatable content, vivid animation of virtual therapist, flexibility (could engage anytime), novelty of the format, emotional safety due to reduced fear of stigma. - Barriers to use of VA-led psychotherapy: preference for human interaction, doubt about effectiveness of VAs, lack of emotional depth and perceived empathy from the VA.   Primary outcomes (readiness and motivation):   - Significant increase in readiness for change and motivation to engage in treatment scores post-treatment (p<0.001, Cohen’s *d* = 0.60) - Significant improvement on motivation for abstaining from drugs score post-treatment (p = 0.045, Cohen’s *d* = 0.30)   Secondary outcomes (self-reported methamphetamine craving, perceived importance and confidence in abstinence):   - Significant decrease in craving (p = 0.01, Cohen’s *d* = 0.38); significant increases in importance of abstaining (p < 0.001, Cohen’s *d* = 0.50) and confidence in abstaining from methamphetamine (p = 0.005, Cohen’s *d* = 0.45) |
| **Virtual Reality** | | | | | | |
| Wang et al., 2019 (Study 1 only)    Li et al., 2023 (Within-subject pre-post intervention component only) |  |  | VR (VRCP) videos designed to pair methamphetamine-related cues with negative consequences; 6 x 5-min sessions over 3 weeks (2 sessions per week) | RCT (wait-list control group)  Experimental pre-post | 61 (men) detoxification centre inpatients;  (1) met criteria for methamphetamine dependance according to DSM-IV, (2) no evidence of current or previous central nervous system disease, (3) no history of cardiovascular diseases, (4) no other DSM-IV Axis I disorders, (5) aged 18 and over  29 (men) rehabilitation centre inpatients;  (1) diagnosis of methamphetamine dependance according to DSM-IV, (2) aged 18 and over | Methamphetamine-related outcomes:   - Participants in the intervention group showed significantly greater reductions in craving (F = 23.2, p<0.001) and liking (F = 21.3, p<0.001) than those in the control-group at follow-up - No significant differences between group on methamphetamine use intention, but bother groups showed a significant reduction over time (p<0.001).   Other outcomes:   - Significant decrease in heart rate variability indices (suggesting diminished physiological reactivity to craving) was observed in the intervention group compared to controls at follow-up (F values ranging from 16.4 to 17.7, all p<0.001)   Methamphetamine-related outcomes:   - Significantly decreased craving (t = 8.94, p<0.001), liking (t = 5.55, p<0.001) and intention to use (t = 7.61, p<0.001) post-intervention   Other outcomes:   - Significantly decreased gamma activity globally (F = 19.63, p<0.001) under drug-cue exposure post-VRCP compared to pre-VRCP (suggesting reduced cue-induced neural activity) including region-specific reductions. |

CBT = Cognitive Behavioural Therapy; CM = Contingency Management; CRA = Community Reinforcement Approaches; VR = Virtual Reality; EMA = Ecological Momentary Assessment; MAUD = Methamphetamine Use Disorder; TAU = Treatment as usual; VRCP = Virtual Reality Counter-conditioning Procedure
